# YKL-40 serum levels are predicted by inflammatory state, age and diagnosis of idiopathic inflammatory myopathies

**DOI:** 10.1038/s41598-023-46491-4

**Published:** 2023-11-06

**Authors:** Mónica Vázquez-Del Mercado, Felipe Pérez-Vázquez, Ana L. Márquez-Aguirre, Erika-Aurora Martínez-García, Efrain Chavarria-Avila, Carlos G. Ramos-Becerra, Andrea Aguilar-Vázquez, Marisol Godínez-Rubí, Beatriz-Teresita Martín-Márquez, Livier Gómez-Limón, Guillermo Márquez-De-La-Paz, Edy-David Rubio-Arellano, Oscar Pizano-Martinez

**Affiliations:** 1 Departamento de Biología Molecular y Genómica, Instituto de Investigación en Reumatología y del Sistema Músculo-Esquelético (IIRSME), Centro Universitario de Ciencias de la Salud, Universidad de Guadalajara, Sierra Mojada No. 950, Puerta 7, Edificio P, Planta Baja. CP 44340, Colonia Independencia, Guadalajara, Jalisco Mexico; 2 Servicio de Reumatología SNP 004086 CONAHCyT, División de Medicina Interna del Hospital Civil Dr. Juan I. Menchaca, Universidad de Guadalajara, Guadalajara, Jalisco Mexico; 3 Cuerpo Académico Inmunología y Reumatología UDG-CA-703, Centro Universitario de Ciencias de la Salud, Universidad de Guadalajara, Guadalajara, Jalisco Mexico; 4 Departamento de Disciplinas Filosófico, Metodológicas e Instrumentales, Centro Universitario de Ciencias de la Salud, Universidad de Guadalajara, Guadalajara, Jalisco Mexico; 5 Unidad de Biotecnología Médica y Farmacéutica, Centro de Investigación y Asistencia en Tecnología y Diseño del Estado de Jalisco (CIATEJ), A.C., Guadalajara, Jalisco Mexico; 6 Departamento de Fisiología, Centro Universitario de Ciencias de la Salud, Universidad de Guadalajara, Guadalajara, Jalisco Mexico; 7 Laboratorio de Mecánica Vascular, Departamento de Fisiología, Instituto de Terapéutica Experimental y Clínica, Centro Universitario de Ciencias de la Salud, Universidad de Guadalajara, Guadalajara, Jalisco Mexico; 8 Doctorado en Ciencias Biomédicas, Centro Universitario de Ciencias de la Salud, Universidad de Guadalajara, Guadalajara, Jalisco Mexico; 9 Laboratorio de Patología Diagnóstica e Inmunohistoquímica, Centro de Investigación y Diagnóstico en Patología, Departamento de Microbiología y Patología, Centro Universitario de Ciencias de la Salud, Universidad de Guadalajara, Guadalajara, Jalisco Mexico; 10 Departamento de Morfología, Centro Universitario de Ciencias de la Salud, Universidad de Guadalajara, Guadalajara, Jalisco Mexico; 11 Departamento de Fisiología, Instituto de Terapéutica Experimental y Clínica, Centro Universitario de Ciencias de la Salud, Universidad de Guadalajara, Guadalajara, Jalisco Mexico

**Keywords:** Idiopathic inflammatory myopathies, Rheumatoid arthritis

## Abstract

YKL-40 increase according to the aging process, and its functions have been associated with tissue remodeling and systemic inflammation. In Rheumatoid Arthritis (RA) it has been proposed as a possible biomarker of activity and severity, however; in the field of idiopathic inflammatory myopathies (IIM) the role of YKL-40 in IIM is not clear. Thus, we aimed to evaluate if there is an association between the serum levels and muscle tissue expression of YKL-40 with age, IIM phenotype, muscle strength and myositis disease activity. The main finding was that age is the most important variable that affects the YKL-40 serum levels. In muscle biopsy, we observed that YKL-40 is mainly expressed in infiltrating lymphoid cells than in muscle tissue. Using ANCOVA according to the b-coefficients, YKL-40 serum levels are predicted by inflammatory state, age, and IIM diagnosis.

## Introduction

YKL-40, also known as human cartilage glycoprotein 39 or chitinase-3 like protein 1, contains highly conserved chitin-binding domains. It lacks chitinase or hydrolase activity due to the exchange of a glutamic acid residue for leucine in the chitinase-3-like catalytic domain^1^. Although the study of this protein began two decades ago, normal serum values vary among the healthy population worldwide. A pivotal YKL-40 study reported that the mean concentration in sera was 43 µg/L in HI between 7 to 45 years old and remains stable for at least 10 years. However, the aging process, increases YKL-40 serum levels^2,3^.

The main sources of YKL-40 are chondrocytes, synoviocytes, endothelial cells, smooth muscle cells and immune cells including monocytes, macrophages, or neutrophils^4,5^. Its function is related to tissue remodeling and/or systemic inflammation. Evidence has pointed out a role as a possible biomarker of activity and severity in some autoimmune rheumatic diseases, mainly rheumatoid arthritis (RA)^6–10^.

In the field of idiopathic inflammatory myopathies (IIM), also known as myositis, the role of YKL-40 in the establishment of the IIM is not clear, few studies have reported a possible association. Elevated YKL-40 expression was found in alveolar macrophages from patients with dermatomyositis /polymyositis (DM/PM) and interstitial lung disease (ILD)^11^. In DM, one of the main histological characteristics, is the complement-mediated microangiopathy that might be related to endothelial damage and YKL-40 expression^12^. In anti-synthetase syndrome (ASyS) YKL-40 expression correlates with TNF-α concentration, arguing that the main source of YKL-40 is the muscle infiltration of inflammatory cells^9,13^.

Our aim was to evaluate if there is an association between the serum levels and muscle tissue expression of YKL-40 with clinical variables such age, IIM phenotype^14^, muscle strength trough Manual Muscle Testing 8 (MMT8)^15^, the clinical activity and disease damage using the myositis disease activity assessment (MYOACT) and muscle damage index (MDI), respectively^16^.

## Results

### Demographic, clinical and laboratory data of RA and IIM patients

**Figure 1:**
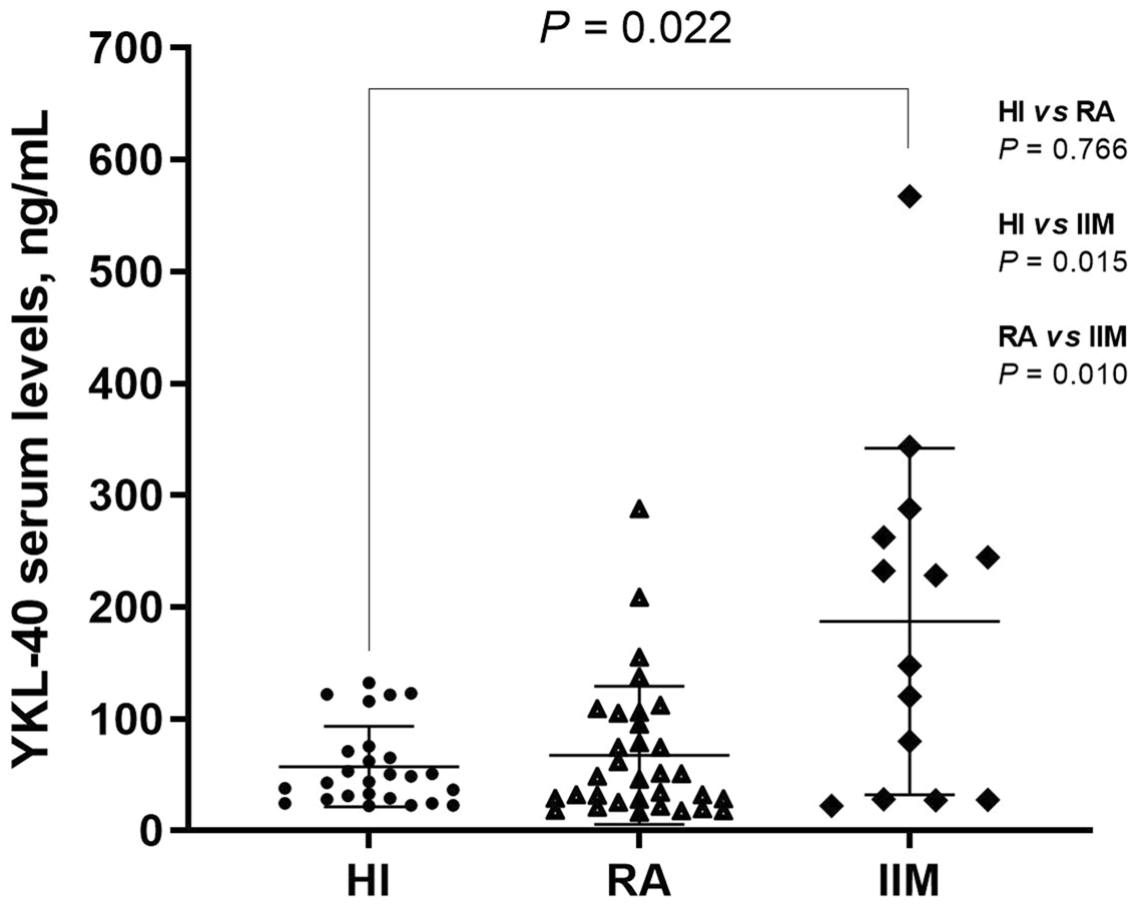
Comparison of YKL-40 serum levels according to study groups. *HI* healthy individual, *RA* rheumatoid arthritis, *IIM* Idiopathic Inflammatory Myopathies, *YKL-40* Chitinase-3-like protein 1. Values were represented as median with ranges. Comparisons were done with Mann–Whitney U and Kruskal–Wallis for independent samples.

Table 1 shows a significant difference in serum levels of YKL-40 between RA and IIM. To confirm that YKL-40 serum levels are influenced by disease process, we compared all study groups: IIM, RA and HI. The most important finding was the difference in IIM against RA (Fig. 1). The clinical features of each IIM patient are shown in Table 2.

**Table 1:** Demographic, clinical and laboratory characteristics of patients with RA and IIM.

	RA	IIM	*P*
	n = 32	n = 14	
Age, yrs	49.27 ± 9.912	44.08 ± 18.302	0.332
Disease duration, yrs	4.99 (2.48 a 7.19)	5.92 (0.79 a 11.52)	0.886
BMI, kg/m^2^	27.55 ± 3.992	28.60 ± 3.519	0.402
ESR, mm/h	22.31 ± 13.338	34.85 ± 29.017	0.157
CRP, mg/L	7.45 (3.40 a 12.15)	4.60 (1.30 a 25.00)	0.732
DAS-28 CRP, score	3.52 ± 1.579	–	–
cfPWV, m/s	7.63 ± 1.057	8.69 ± 3.041	0.259
pSBP, mmHg	113.45 ± 8.656	124.50 ± 20.034	0.088
pDBP, mmHg	70.07 ± 6.589	82.83 ± 16.749	0.024
pMBP, mmHg	84.53 ± 6.434	96.72 ± 17.306	0.035
MMT8, score	NA	80.00 (76.75 a 80.00)	–
YKL-40, ng/mL	46.82 (25.50 a 101.96)	187.80 (27.87 a 268.43)	0.010

Values were represented as mean ± SD and median with ranges. Comparisons were done with the Student’s T test and Mann–Whitney U for dependent samples.

*RA* rheumatoid arthritis, *IIM* Idiopathic Inflammatory Myopathies, *yrs* years, *BMI* body mass index, *ESR* erythrocyte sedimentation rate, *CRP* C-reactive protein, *DAS-28 CRP* disease activity score on 28 joints. *cfPWV* carotid to femoral pulse wave velocity, *pSBP* peripheral systolic blood pressure, *pDBP* peripheral diastolic blood pressure, *pMBP* peripheral mean arterial pressure, *MMT8* Manual Muscle Testing 8, *YKL-40* Chitinase-3-like protein 1, *NA* not applicable.

**Table 2:** Clinical features of IIM patients.

Patient	Age	Gender	Presence of MSA/MAA	IIM subgroup	Disease duration (years)	Comorbidities	MMT8 score (0—80)	MYOACT score	MDI score	ESR (mm/h)	PCR (mg/L)	Serum levels of muscle enzymes	YKL-40 serum levels (ng/mL)	Treatment	Clinical status
6001	38	Male	Anti-Mi-2α, anti-Mi-2β, anti-Ku	DM	8.36	–-	80	0.00	0.00	3.0	1.3	CPK = 305.0 U/L; AST = 25.7 U/L; ALT = 48.2 U/L	21.98	Prednisone, Hydroxychloroquine, Azathioprine	Remission, lack of physical rehabilitation
6002	20	Male	Anti-MDA5, anti-Ku	ADM	0.06	–	80	0.03	0.00	30.0	< 6.0	CPK = 42.0 U/L; AST = 28.0 U/L; ALT = 61.0 U/L; DHL = 570.0 U/L	28.04	Prednisone, Hydroxychloroquine, Azathioprine	Remission
6003	70	Female	Anti-EJ	PM + ASS	0.84	–	80	0.03	0.02	10.0	0.1	CPK = 33.0 U/L; AST = 12.0 U/L; ALT = 11.0 U/L; DHL = 50.0 U/L; aldolase = 1.0 U/L	79.61	Methotrexate, Prednisone, Mycophenolate	Mild activity
6004	36	Male	Seronegative	DM	5.92	–	76	0.02	0.01	9.0	7.4	CPK = 254.0 U/L: AST = 49.0 U/L; ALT = 118.0 U/L; DHL = 167.0 U/L; aldolase = 8.6 U/L	120.05	Methotrexate, Prednisone	Mild activity
6006	49	Male	Anti-Mi-2α, anti-Mi-2β	DM	2.34	–	52	0.03	0.02	76.0	–	CPK = 2888.0 U/L	244.43	Prednisone, Azathioprine, Omeprazole	Moderate activity
6007	20	Female	Anti-TIF-1γ	DM	0.62	–	80	0.00	0.00	36.0	4.6	CPK = 63.0 U/L; AST = 30.1 U/L; ALT = 12.8 U/L; DHL = 349.0 U/L; aldolase = 4.9 U/L	27.26	Methotrexate, Prednisone, Hydroxychloroquine	Remission
6008	79	Male	Seronegative	PM and gastric cancer	0.54	Diabetes mellitus 2, hypertension	77	0.03	0.00	36.0	4.0	CPK = 184.0 U/L; AST = 35.6 U/L; ALT = 54.1 U/L; DHL = 230 U/L; aldolase = 10.6 U/L	343.08	Methotrexate, Prednisone	Remission
6010	33	Female	Anti-NXP2, anti-SRP	DM	25.80	–	80	0.00	0.00	34.0	0.6	CPK = 54.0 U/L; AST = 15.0 U/L; ALT = 31.0 U/L; DHL = 336.0 U/L; aldolase = 1.0 U/L	27.33	Methotrexate, Prednisone, Hydroxychloroquine	Remission
6011	65	Female	Anti-SRP	DM	5.92	Diabetes mellitus 2, hypertension, osteoporosis	71	0.05	0.07	44.0	4.5	CPK = 75 U/L	287.59	Methotrexate, Prednisone, Gabapentin	Mild activity
6012	46	Female	Seronegative	PM	22.20	Interstitial lung disease, diabetes mellytus 2, mixed anxiety and depression disorder, osteroporosis	73	0.00	0.00	43.0	27.0	CPK = 46.0 U/L; AST = 14.0 U/L; ALT = 15.0 U/L; DHL = 128 U/L	232.38	Hydroxychloroquine, Azathioprine, Gabapentin, Escitalopram	Mild activity
6013	34	Male	Anti-NXP2, anti-PMScl-100	Scleromyositis	7.87	Gout	80	0.03	0.00	16.0	24.0	AST = 25.0 U/L; ALT = 75.0 U/L; aldolase = 6.5 U/L	262.04	Prednisone, Mycophenolate, Diltiazem, Allopurinol, Colchicine	Remission
6015	22	Female	Anti-Mi-2β	DM	1.71	–	70	0.01	0.01	8.0	2.0	CPK = 170.0 U/L; DHL = 251.0 U/L; aldolase = 3.2 U/L	147.43	Methotrexate, Prednisone	Mild activity, lack of physical rehabilitation
6018	48	Female	Seronegative	DM	18.33	Rheumatoid arthritis	80	0.02	0.00	95.0	100.0	CPK = 217.7 U/L; AST = 28.1 U/L; ALT = 24.9 U/L; DHL = 410.0 U/L; aldolase = 0.6 U/L	228.17	–	Remission
6019	50	Female	Seronegative	PM and cervical cancer resolved	9.25	–	80	0.00	0.00	89.0	25.0	CPK = 71.0 U/L; AST = 25.0 U/L; ALT = 13.0 U/L; DHL = 162.0 U/L	566.87	Azathioprine	Remission

### YKL-40 serum levels in IIM are associated with age, but not with disease duration

**Figure 2:**
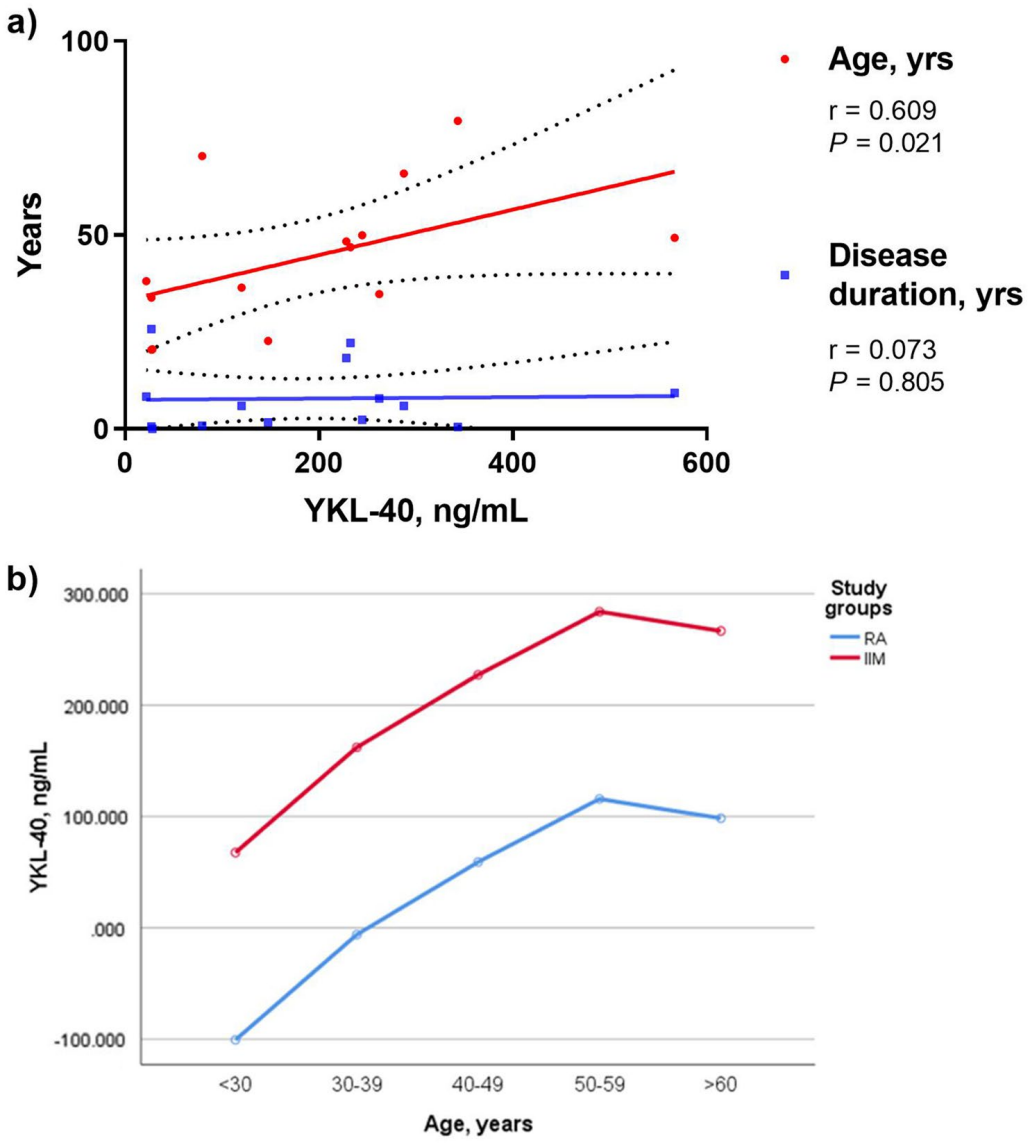
YKL-40 serum levels according to age in RA and IIM. (**a**) Correlation between age and disease duration with YKL-40 serum levels of IIM and (**b**) mean YKL-40 by decade in study groups. Predicted mean YKL-40 serum levels according to age group and study group. RA = rheumatoid arthritis, IIM = Idiopathic Inflammatory Myopathies. Predicted means were analyzed by ANCOVA.

To assess which variables can determinate higher YKL-40 serum levels in IIM, we evaluated the correlation between YKL-40 serum levels and clinical variables (Supplementary Table 1). We found that age is most important variable that affects the YKL-40 serum levels but not the duration of disease in IIM (Fig. 2a).

### Predictive value of in CRP, age and diagnosis on serum levels of YKL-40

To evaluate the influence of inflammatory state (CRP), age and IIM diagnosis on YKL-40 serum levels, we analyze these variables by ANCOVA. According to the b-coefficients, YKL-40 serum levels were increased with inflammation, age and IIM diagnosis mainly (Table 3). It is interesting to denote although the behavior of YKL-40 serum levels by decades is similar between both groups IIM patients shown higher levels at the same age (Fig. 2b).

**Table 3:** Multiple linear regression analysis for YKL-40 serum levels (ng/mL).

Total R^2^	0.422		
	β-Coefficient (95% CI)	*P*	
Constant	− 218.598	(− 364.69 a–72.51)	0.004
Age, years	3.031	(0.82 a 5.24)	0.008
Patient group*	119.930	(55.28 a 184.58)	0.001
CRP, mg/L	1.864	(0.112 a 3.62)	0.038

β-Coefficient are given as value (95% confidence Interval); unit for β-Coefficient is ng/mL). The method forward was used with P_IN_ = 0.05 and P_OUT_ = 0.10). Variables included from the model Age (years), C-reactive protein (CRP) and patients’ group (*IIM* Idiopathic Inflammatory Myopathies, *RA* rheumatoid arthritis).

*Patient group is equal to: 1 when RA; 2 when IIM).

### YKL-40 expression in muscle tissue from IIM patients

**Figure 3:**
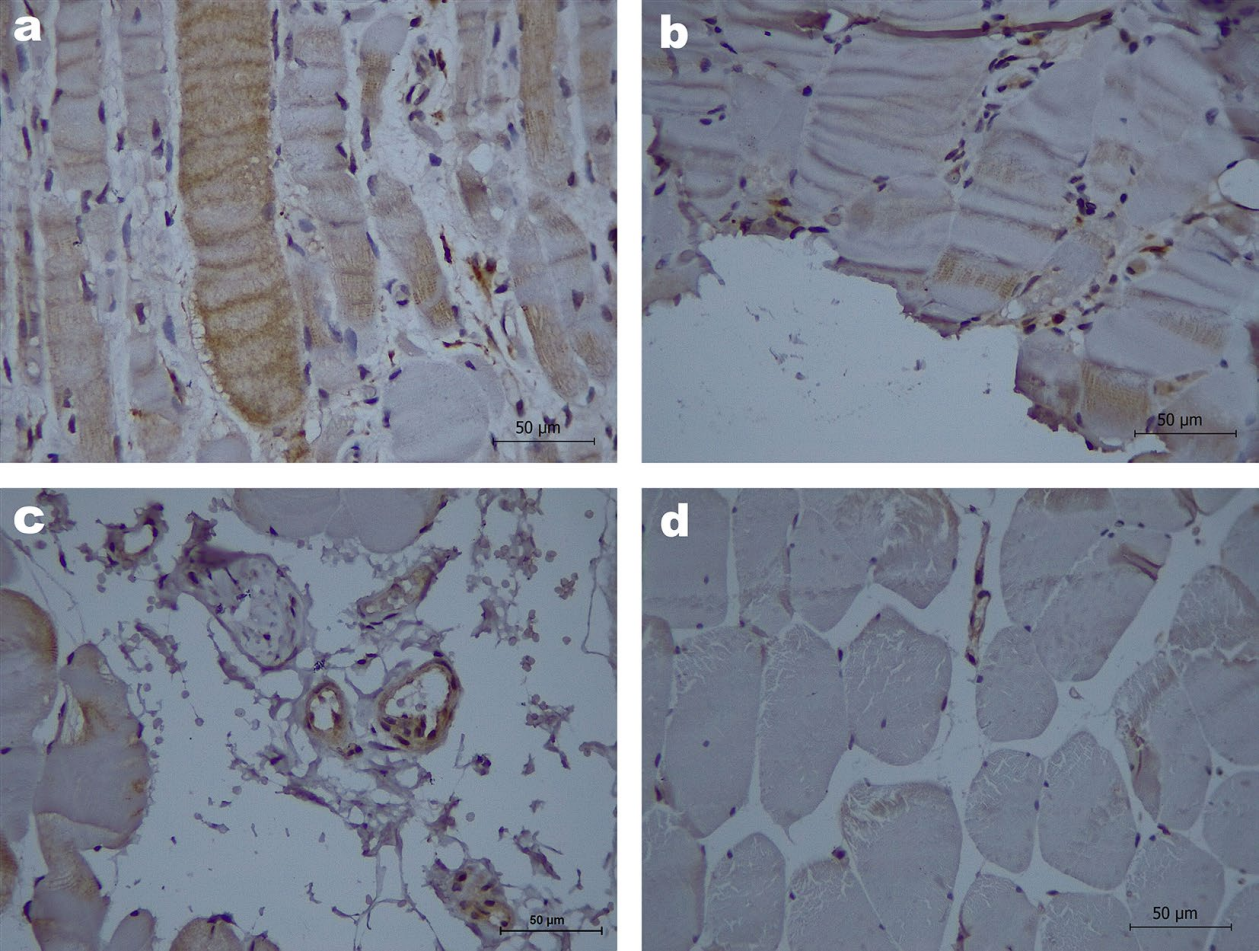
Protein expression of YKL-40 in MII patients muscle samples evaluated by immunohistochemistry. (**a**) Polymyositis patient, anti-PL12 positive, MMT8 = 56, MYOACT = 0.33, MDI = 0.28; moderate cytoplasmatic expression (2+) multifocal in lymphocytes located in endomysium and perimysium as well as in lymphocytes surrounding blood vessels in the perimysium. Some muscular fibers with atrophic appearance and evident inflammatory damage showed YKL-40 expression from mild to moderate, the endothelium also showed moderate cytoplasmatic expression in multiple blood vessels. (**b**) Polymyositis patient, anti-EJ and anti-Ro-52 seropositive, MMT8 = 135, MYOACT = 0.28, MDI = 0.12; mild and multifocal cytoplasmatic expression (1+) in scarce lymphocytes in the endomysium and surrounding blood vessels in the perimysium; muscular fibers without evidence of YKL-40 expression. (**c**) Amyopathic dermatomyositis patient, anti-MDA5 seropositive, MMT8 = 150, MYOACT = 0, MDI = 0; mild and focal cytoplasmatic expression (1+) in scarce lymphocytes in the endomysium, muscular fibers without evidence of YKL-40 expression. (**d**) Dermatomyositis patient, anti- TIF-1γ seropositive, MMT8 = 150, MYOACT = 0, MDI = 0; without YKL-40 expression in muscle or lymphoid cells.

In addition to quantifying YKL-40 serum levels, we were able to obtain 17 muscle tissue samples from myositis patients, to evaluate YKL-40 in situ protein expression. We rather observed YKL-40 expression in infiltrating lymphoid cells (11 patients, 61.1%) than muscle tissue (one patient, 5.6%), mainly in endomysium. The most representative images in decreased order of YKL-40 expression are shown in Fig. 3a–d.

In panel a, we show the only positive patient in muscle tissue. It is important to denote that this patient had the lowest MMT8 along the highest MYOACT and MDI scores.

**Figure 4:**
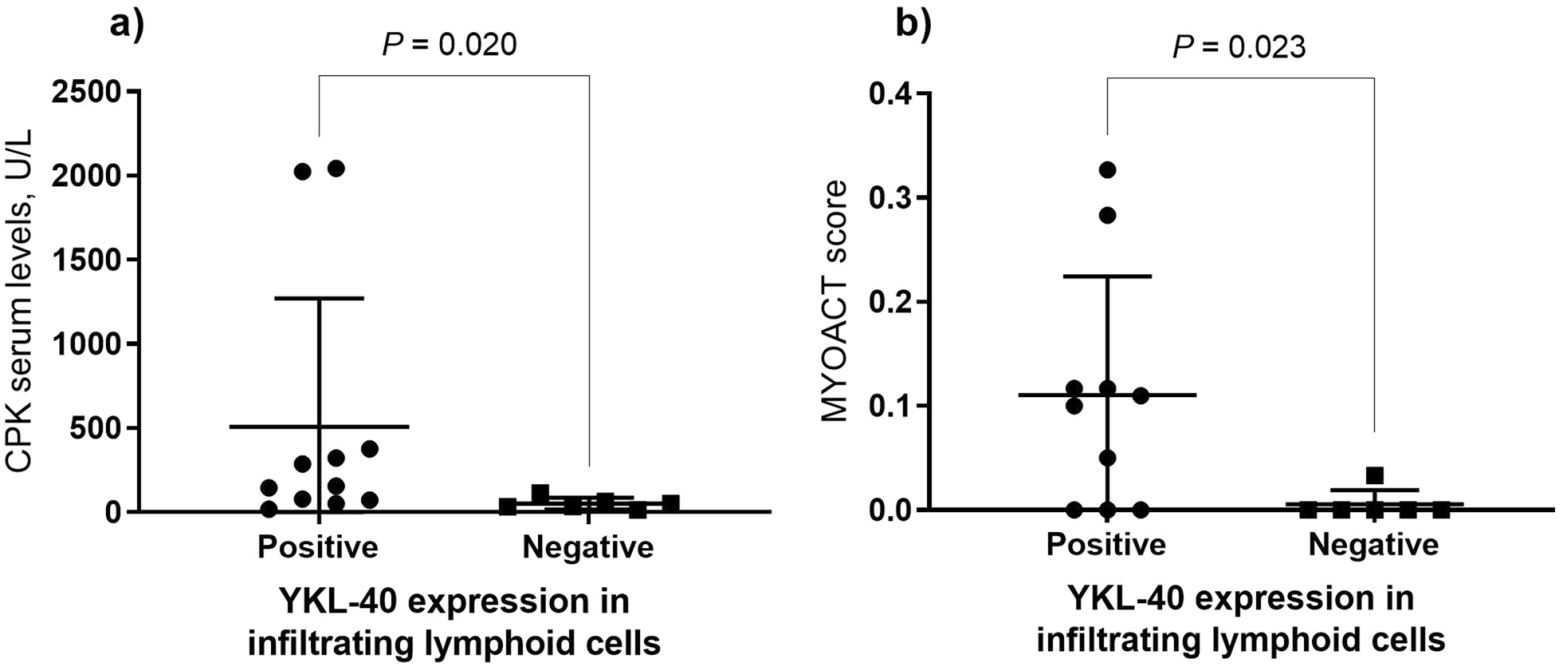
Clinical and biochemical parameters according to positivity for expression of YKL-40 in infiltrating lymphoid cells. (**a**) Higher CPK serum levels in patients positive for YKL-40 expression in infiltrating lymphoid cells and (**b**) higher MYOACT score in patients positive for YKL-40 expression in infiltrating lymphoid cells. *CPK* creatine phosphokinase, *MYOACT* Myositis disease activity assessment visual analogue scale. Comparisons were done with Mann–Whitney U test with Fisher’s Exact test.

We did not observe any association between YKL-40 protein expression and the presence of MSA/MAA or the clinical phenotype, however; we found that positives patients for YKL-40 expression in infiltrating lymphoid cells had higher CPK serum levels (506.4 ± 763.70 vs 51.33 ± 34.67, *P* = 0.020) as well as higher MYOACT score (0.1 ± 0.11 vs 0.0 ± 0.01, *P* = 0.023) (Fig. 4). These results denote there is a lack of knowledge about the clinical relevance of YKL-40 expression in muscle tissue and lymphoid cells.

## Discussion

In this study, YKL-40 serum levels are influenced by factors such as age, inflammation, and diagnosis of autoimmune diseases (RA/MII)^3^. Currently, due to its participation in tissue remodeling and degradation, attempts have been made to use it as a biomarker in proinflammatory states as well as to be an indicator of poor prognosis in inflammatory diseases^6,7^. However, its usefulness is still controversial because the full biological effects are still unknown. Moreover, the specific factors that promote its expression as well as its interaction with the majority of the cytokines and molecules involved in the development and establishment of autoimmune inflammatory diseases are not well established^17^.

It has been described that YKL-40 serum levels are increased with age, in various cardiovascular, metabolic, and systemic inflammatory diseases^3^. Bojesen et al. found that the serum values of YKL-40 increased exponentially with aging. In subjects with two YKL-40 measurements 10 years apart, the mean increase in YKL-40 was 1.5 μg/L/year^18^. Regarding inflammatory diseases, in anti-neutrophil cytoplasmic antibody (ANCA)-associated vasculitis, increased serum levels of YKL-40 were reported, hypothesizing that might have a role to promote chemotaxis, tissue damage, and vascular injury^19^.

In RA, it has been recognized as a potential candidate autoantigen, additionally is produced, and secreted by monocytes differentiated into macrophages, articular chondrocytes, synovium, peripheral blood mononuclear cells (PBMCs) and endothelium in these patients^17,19,20^. It has been proposed that the pathogenic mechanism of YKL-40 in RA initiates with its binding to HLA-DR4 peptide-binding motif promoting the mononuclear cells proliferation and HLA-DM plays a key role to presenting YKL-40 to CD4 + T cell. In addition, antigen presenting cells (APCs) present YKL-40 in sites where the RA is in early phase, indicating an association for YKL-40 in RA pathogenesis^20–22^. On the other hand, differentiated DR4 + dendritic cells and macrophages are similar to synovial joint APCs and have the potential to perform the MHC II-presentation of YKL-40 epitopes resulting in higher levels in synovial and serum^21,22^. Although the pathogenic mechanism in RA has been elucidated, the agents that promote the expression of YKL-40 in RA are still missing, it has been associated with the development of a chronic, destructive, relapsing arthritis due to its role in tissue remodeling and degradation. It is considered as an effective marker in estimating RA disease activities, prognostic value and may be a therapeutic target^19^.

Regarding IIM, the information is even more limited because few studies have been carried out in this regard thus the role played by YKL-40 in this field has not yet been established. Regarding its proinflammatory action and relationship with diagnosis and phenotype of IIM, Noguchi et al. found significantly elevated serum in patients with PM/DM compared to the healthy population, as well as age-corrected serum YKL-40 values were significantly increased in patients with PM/DM compared to the HC. In muscle biopsies found infiltration of YKL-40-positive inflammatory cells (probably macrophages) in the endomysium and perimysium this suggest that cells other than CD8 + and CD4 + T cells may cause inflammation^23^.

Ming-Zhu Gao et al. measured YKL-40 levels in patients with DM/PM, and HI and reported significantly higher levels in patients with IIM compared to the control group (51.6 vs 27.8 ng/mL, respectively)^6^. In a systematic review by Cui et al. reports levels of 84.09 ng/mL in patients with PM and DM vs 27.37 ng/mL in HI^24^. On the other hand, Carboni et al. analyzed YKL-40 serum levels and its expression in muscle tissues in patients with ASSD. However, YKL-40 serum levels did not correlate with other clinical, laboratory, disease status, or therapeutic parameters, moreover, YKL-40 was expressed by the inflammatory cells of the muscle tissue^13^. Our study strengthens these results with serum levels of 187.80 ng/mL in patients with IIM vs 46.82 ng/mL with RA and 57.17 ng/mL in HI as well as their presence mainly in inflammatory cells. This elevation could be explained by inflammation, macrophage activation, fibroblast destruction, and vascular changes present.

As mentioned earlier, several biological effects of YKL-40 are known such as inflammation and tissue remodeling as well as its main sources, highlighting its exacerbated expression in inflammatory diseases such RA or SLE, however; some researchers has pointed out that this expression can vary according disease type which could be due to the multiorgan damage in IIM compared with RA where the damage is directed against joints mainly^19,24^. Tang et al. found that YKL-40 concentration was significantly higher in IIM patients with myocardial injury than without myocardial injury^25^.

In addition, YKL-40 has a role in cardiovascular diseases such as early atherosclerosis, essential hypertension, and other progressing vascular complications. In IIM patients as in many others autoimmune diseases the serum levels of this protein have a positive association, specifically with atherosclerosis and could predict both overall and cardiovascular mortality^5,26,27^.

We aimed to know if YKL-40 are influenced by some factors in IIM such as was mentioned by Tizaoui et al., first, we compared some demographic, laboratory and clinical variables between patients with RA and IIM mentioned in Table 1. Of all the variants analyzed, we found a significant difference in pDBP (P = 0.024) and pMBP (P = 0.035) being higher in IIM patients. This could be due to that blood vessels suffer damage in early stages of inflammatory diseases development, which alters the blood pressure and increases the risk of cardiovascular damage^28,29^. Moreover, the endothelial alterations in IIM develops into microangiopathy, causing blood pressure alterations^12^. Although information about how the process occurs precisely is scarce, it was pointed out that cardiovascular disease (CVD) and cardiovascular risk are increasing the mortality in IIM patients, but this becomes controversial because recently one single-center cross-sectional study reported that CV risk factors in IIM patients are not significant compared to HI but they are in IIM when are associated with age, disease duration, duration of therapy and body composition, which could be related with our patients enrolled in our study^25,30^. Although these variables evaluated were significant between these two groups, the serum levels of YKL-40 were the most significant variable (P = 0.010), being higher in IIM than in RA patients (187.80 ng/mL *vs* 46.82 ng/mL, respectively).

Once we established that IIM presents higher serum levels of YKL-40 than RA, we investigated if YKL-40 serum levels were affected by age or disease duration. Our results show that only aging has a positive correlation with increased YKL-40 but no with disease duration. The reports carried out by Johansen in 2006 and Schultz 2010 demonstrated clearly that aging is predictive for increases YKL-40 in HI, but in our IIM patients the concentration of this protein are more elevated due to inflammation and multiorgan damage^2,3^.

We evaluated the predictive value of inflammatory state, age and diagnosis of IIM on serum levels of YKL-40 and elucidated that CRP has predictive value on YKL-40 serum levels in IIM patients (*P* = *0.038*) which is according with a cross-sectional study and systematic review published by Cui and *et. al*. In addition, age and the IIM diagnosis (*P* = *0.008* and *P* = *0.001*, respectively) showed to be powerful predictors on YKL-40 serum levels^24^. On the other hand, we confirmed that age and IIM diagnosis influences the YKL-40 concentration importantly because YKL-40 serum levels are the highest when are compared against control and RA groups allowing us to know that the presence of the disease or its type influence the YKL-40 concentration. Some reports refer that YKL-40 concentration in HI are stable for many years but increase with aging or inflammatory conditions. Other researchers reported that normal YKL-40 serum levels can be different among healthy population, therefore, they recommend that baseline values should be established for each study because in addition to environmental factors, the genetic load is other variable that can influence the pattern of expression of this protein^26^. We mentioned previously the pathogenic mechanism by which the expression of YKL-40 is mediated in RA and its possible role in IIM, but the information is not yet enough.

Regarding the in-situ analysis of YKL-40 muscle expression, we observed YKL-40 is mainly expressed in inflammatory cells rather than muscle cells and we also observed YKL-40 expression is associated with higher CPK serum levels and MYOACT score that are commonly related to higher inflammation and muscle weakness. This observation is in accordance with the unique previous report of YKL-40 in muscle tissue made by Carboni et al.^13^, thus, it supports its role in inflammation, as well as its function as a clinical marker of poor prognosis in inflammatory diseases.

Considering all our findings, we showed the expression of YKL-40 in HI as well as in patients with RA and IIM and the possible factors that could influence its expression.

## Conclusions

YKL-40 serum levels are predicted by inflammatory state, age and IIM diagnosis.

## Methods

We performed a non-probabilistic sampling type study to evaluate the association of YKL-40 in IIM patients and as a control groups healthy individuals (HI) and RA patients.

### Approval by the ethics and research committees

All individuals gave their written consent before enrollment. The protocol was approved by both ethics and research committees from Hospital Civil Dr. Juan I. Menchaca (CONBIOETICA-14-CEI-008-20161212) and (17CI14039116 COFEPRIS) respectively, as well as registered by Secretaria de Salud del Estado de Jalisco (registration code 0501/21HCJIM2021). Research was conducted following Helsinki criteria according to its last updated in 2013, Fortaleza, Brazil.

### Individuals

Patients were recruited from the rheumatology service at the Hospital Civil “Dr. Juan I. Menchaca” from Guadalajara, Jalisco, México. The inclusion criteria were age > 18 to < 80 years old, without known comorbidities such as cancer, cardiovascular disease, diabetes melllitus. Exclusion criteria were current pregnancy.

The study group was composed by 17 subjects classified as IIM according to the EULAR/ACR 2017 criteria^14^, 32 subjects classified as RA according to EULAR/ACR 2010^31^, as well as 26 HI as control group. Informed written consent was signed by every patient and subject before serum and muscle samples were obtained.

### Clinical assessment

Each patient was interviewed using a structured questionnaire to gather demographic and clinical variables (body mass index [BMI], carotid femoral pulse wave velocity [cfPWV], peripheral systolic blood pressure [pSBP], peripheral diastolic blood pressure [pDBP] and peripheral mean arterial pressure [pMBP]). The IIM clinical assessment was evaluated trough the clinical tools MMT8, MYOACT and MDI^15,16^.

In the case of RA patients, the activity index was evaluated by the disease activity score on 28 joints^32,33^.

### Serum samples and laboratory measurements

Serum samples were aliquoted and stored at − 80 °C until used. Laboratory measurements included erythrocyte sedimentation rate (ESR) measured using Wintrobe’s method^34^ as well as the C-reactive protein (CRP) determined by nephelometry. Muscular enzymes serum levels (CPK, AST, ALT, LDH and aldolase) were recorded at the time of diagnosis and recruitment.

### Determination of YKL-40 serum levels

The YKL-40 serum levels were quantified using the YKL-40 HUMAN ELISA KIT (MBS824919, My BioSource, Inc. P.O. BOX 153308 San Diego, CA 92195-3308 USA) according with the instructions of the manufacturer. The kit allows quantification of human YKL-40 protein within the range of 62.5–4000 pg/ml.

### Immunohistochemistry

YKL-40 protein expression was evaluated from quadriceps muscle biopsies using heat induction epitope retrieval technique. Briefly, the paraffin embedded muscle tissue was deparaffinized with xylol baths for 10 min, later hydrated with alcohol-based solutions in decreasing order, distilled water, citrate buffer pH 6.0, and blocked with H_2_O_2_ at room temperature; samples were incubated with PBS, primary antibody (ab77528, ABCAM) and secondary antibody to reveal with diaminobenzidine, followed by washings and microscopic observation.

### Statistical analysis

According to the type of variable and the distribution of the data, the results are presented as frequency, median-interquartile range and standard deviation-mean. The associations were made using univariate/multivariate analysis, parametric quantitative analysis was made applying Student's t-test, non-parametric variables were analyzed with the Spearman´s correlation or Mann–Whitney U test. The statistical program SPSS v.24® was used. *P*-value less than 0.05 was defined as statistical significance.

### Supplementary Information

Supplementary Table 1.

## Data Availability

The original images from microscope showed on Fig. 3 are available in the FigShare repository: https://doi.org/10.6084/m9.figshare.23601135. The raw data is available in the FigShare repository: https://doi.org/10.6084/m9.figshare.23930172.
