# Supplementary material for: YKL-40 serum levels are predicted by inflammatory state, age and diagnosis of idiopathic inflammatory myopathies
Source: Sci Rep. 2023 Nov 6;13:19172. doi: 10.1038/s41598-023-46491-4 (PMC10628093; doi:10.1038/s41598-023-46491-4)
Supplement: Supplementary file 1 — Supplementary Table 1. [file 41598_2023_46491_MOESM1_ESM.docx]

**Supplementary Table 1. Correlation YKL-40 with clinical characteristics of** **autoimmune diseases**

|  | **YKL-40, ng/mL** | | |
| --- | --- | --- | --- |
|  |  | **RA** | **IIM** |
|  | **n = 46** | **n = 32** | **n = 14** |
| Age, yrs | **0.302**  **(0.042)** | 0.322  (0.072) | **0.609**  **(0.021)** |
| Disease duration, yrs | 0.068  (0.652) | 0.045  (0.808) | 0.073  (0.805) |
| BMI, kg/m2 | 0.239  (0.110) | 0.182  (0.318) | 0.270  (0.350) |
| ESR, mm/h | 0.245  (0.104) | 0.121  (0.510) | **0.556**  **(0.049)** |
| CRP, mg/L | **0.327**  **(0.032)** | 0.198  (0.277) | 0.600  (0.051) |
| DAS-28 CRP, score | - | 0.296  (0.100) | - |
| cfPWV, m/s | 0.265  (0.090) | 0.155  (0.413) | 0.385  (0.216) |
| pSBP, mmHg | **0.477**  **(0.001)** | **0.386**  **(0.029)** | **0.592**  **(0.043)** |
| pDBP, mmHg | **0.340**  **(0.024)** | 0.119  (0.518) | 0.343  (0.276) |
| pMBP, mmHg | **0.445**  **(0.002)** | 0.244  (0.178) | 0.515  (0.087) |
| MMT8, score | - | - | -0.380  (0.278) |

**YKL-40 =** Chitinase-3-like protein 1, **RA =** rheumatoid arthritis, **IIM =** Idiopathic Inflammatory Myopathies, **yrs =** years, **BMI =** body mass index, **ESR =** erythrocyte sedimentation rate, **CRP =** C reactive protein, **DAS-28 =** disease activity score on 28 joints, **cfPWV =** carotid to femoral pulse wave velocity, **pSBP =** peripheral systolic blood pressure, **pDBP =** peripheral diastolic blood pressure, **pMBP =** peripheral mean arterial pressure, **MMT8 =** Manual Muscle Testing 8. Correlations were done with Spearman.
